# Effects of obesity‐associated plasma markers on adipose stem cell function and epigenetic regulation

**DOI:** 10.1002/oby.24321

**Published:** 2025-06-16

**Authors:** Andressa França Sousa Bispo, Jussara de Jesus Simao, Miguel Ambrizzi Moraes, Ana Beatriz Marques Abel, Victor Tadeu Gonçalves Plata, Monica Marques Telles, André Valente Santana, Paula Volpe, Lucia Maria Armelin‐Correa, Maria Isabel Cardoso Alonso‐Vale

**Affiliations:** ^1^ Postgraduate Program in Chemical Biology Institute of Environmental Sciences, Chemical and Pharmaceutical, Federal University of São Paulo Diadema Brazil; ^2^ Department of Biological Sciences Institute of Environmental Sciences, Chemical and Pharmaceutical, Federal University of São Paulo Diadema Brazil; ^3^ Postgraduate Program in Nutrition Paulista School of Medicine, Federal University of São Paulo São Paulo Brazil; ^4^ Postgraduate Program in Interdisciplinary Surgical Science Paulista School of Medicine, Federal University of São Paulo São Paulo Brazil; ^5^ Rede D'Or São Luiz Hospitals São Paulo Brazil

## Abstract

**Objective:**

This study investigates the correlations between obesity‐related plasma markers and epigenetic/inflammatory changes in white adipose tissue (WAT), focusing on adipose‐derived stem cells (ASCs). We hypothesize that obesity modulates histone H3K27 marks, modified by demethylases (lysine‐specific demethylase 6A and 6B [KDM6A/KDM6B]) and acetylases (CREB–binding protein [CREBBP]/histone acetyltransferase EP300), affecting ASC function.

**Methods:**

Serum and visceral WAT (omental region) was collected from male patients (*n* = 16, 30–50 years old) undergoing elective gastric or bariatric surgery. BMI and obesity markers were correlated with changes in ASCs (transcript expression, proliferation, and secretion) using reverse transcriptase‐polymerase chain reaction.

**Results:**

ASCs from individuals with higher BMI exhibited slower proliferation, increased inflammatory profile, and reduced adipogenic potential, with lower expression of key adipogenic genes. H3K27 acetylase transcripts were also negatively correlated with adipogenesis regulators. Moreover, C‐C motif chemokine 2 (CCL2) and KDM6A expression was higher in the group with obesity, as were CREBBP and EP300. Finally, leptin levels positively correlated with serum, WAT, and ASC CCL2 expression. In vitro, leptin exposure enhanced CCL2 expression/secretion and increased KDM6A/KDM6B and EP300 transcription.

**Conclusions:**

In vitro leptin exposure enhanced CCL2 expression/secretion and increased KDM6A/KDM6B and EP300 transcription, highlighting how obesity‐driven epigenetic mechanisms, including leptin‐mediated pathways, disrupt ASC plasticity and perpetuate adipose tissue dysfunction, offering novel therapeutic targets for metabolic disease intervention.

**STUDY IMPORTANCE**

**What is already known?**

- Obesity induces epigenetic alterations in adipose‐derived stem cells (ASCs), impairing their function and promoting inflammation in white adipose tissue (WAT).
- Leptin and CCL2 levels are elevated in obesity and correlate with WAT dysfunction and metabolic comorbidities.

**What does this study add?**

- Leptin upregulates CCL2 in ASCs and putative H3K27ac involvement: Leptin increases KDM6A/KDM6B, EP300, and CCL2. Public data suggest H3K27ac enrichment at CCL2, supporting future validation of this epigenetic link.
- Obesity‐linked epigenetic dysregulation impairs ASC function: ASCs from high‐BMI individuals show impaired proliferation, adipogenesis (↓ KLF15, CEBPA, CEBPB), and increased inflammation (↑ CCL2, TNF‐α), correlated with elevated KDM6A, CREBBP, and EP300.

**How might these results change the direction of research or the focus of clinical practice?**

- Targeting epigenetic regulators in ASCs: Our findings identify KDM6A/KDM6B, EP300, and CREBBP as novel therapeutic targets to mitigate obesity‐associated inflammation and restore ASC function, potentially preventing WAT dysfunction.
- Targeting the leptin‐H3K27ac‐CCL2 axis: Disrupting leptin‐induced H3K27ac‐mediated CCL2 activation in ASCs could reduce WAT macrophage infiltration and inflammation.

## INTRODUCTION

Obesity is defined as an excess of body fat in a quantity that is harmful to health and is a progressive and relapsing disease that has reached epidemic proportions. The quickest and most accessible way to diagnose this condition is by calculating the individual's body mass index (BMI), which can be obtained simply by calculating the ratio of weight in kilograms to height in meters squared. If BMI is 30 kg/m^2^ or more, the individual is considered to have obesity (classified as class I obesity [BMI 30–34.9], class II [BMI 35–39.9], and class III [BMI ≥40]), whereas BMI values between 25 and 29.9 are considered overweight, between 18.6 and 24.9 are considered normal (eutrophic), and 18.5 or less is considered underweight [1].

Obesity induces a low‐grade inflammatory environment, characterized by a significant increase in circulating cytokines and endotoxins, which activate the nuclear factor‐κB (NF‐κB) signaling pathway in white adipose tissue (WAT), resulting in tissue dysfunction and the progression of metabolic comorbidities [2, 3]. WAT is distributed in two main depots, subcutaneous WAT (sWAT) and visceral WAT (vWAT), composed mainly of mature adipocytes, and it plays a central role in regulating energy homeostasis. This tissue contains a matrix of connective tissue, nervous tissue, and lymph nodes, which constitute the stromal vascular fraction, a rich source of preadipocytes, endothelial progenitor cells, immune cells like macrophages, and a large number of mesenchymal stem cells known as adipose‐derived stem cells (ASCs). ASCs have high plasticity and the ability to proliferate and differentiate, influencing the metabolic and functional dynamics of WAT. When activated, they can undergo adipogenesis, committing to the adipocyte lineage and differentiating into mature adipocytes, the specialized cells of WAT (reviewed by Mo et al. [4]). Adipogenesis, when controlled, is beneficial as it allows for proper lipid storage, preventing metabolic complications. In individuals with metabolically healthy obesity, the plasticity of WAT remains within controlled limits, leading to a moderate increase in the number and size of adipocytes, with mild inflammation and increased angiogenesis, which helps prevent metabolic disorders [5, 6, 7]. However, when the adaptability of WAT is exceeded, adipocytes become overloaded, leading to cellular dysfunction, inflammation, and insulin resistance [8].

The chemokine ligand CCL2, also known as monocyte chemoattractant protein‐1 (MCP‐1), plays a crucial role by promoting the recruitment of monocytes that accumulate in WAT during obesity [9, 10]. The secretion of CCL2 by adipocytes, along with other adipokines, appears to be more closely associated with obesity‐related risks than other adipokines [9, 11]. Furthermore, studies suggest that preadipocytes and ASCs may be significant sources of proinflammatory mediators, such as CCL2 [12].

Simultaneously, obesity is associated with epigenetic modifications affecting ASCs, particularly in genomic regions regulating adipogenesis. Ejarque et al. [13] demonstrated that, although global DNA methylation is preserved during adipogenesis, obesity predisposes ASCs to dynamic methylation/acetylation changes impairing WAT function and promoting the development of metabolic syndrome. The acetylation of histone 3 lysine 27 (H3K27) plays a critical role in this process, influencing the expression of genes related to adipogenesis and the differentiation of ASCs into mature adipocytes [14, 15, 16].

In this study, we investigated how plasma markers of obesity correlate with alterations in WAT, specifically in ASCs, focusing on epigenetic and inflammatory pathways. Plasma and vWAT from patients undergoing bariatric surgery were analyzed to correlate BMI and obesity‐related plasma markers with vWAT‐derived ASCs. We selected vWAT over sWAT due to its stronger association with metabolic dysfunction, including heightened inflammation and impaired ASC function [13, 17, 18]. Our analyses targeted obesity‐driven epigenetic changes (H3K27 modifications), inflammatory profiles, proliferation/secretion capacity of ASCs, and key adipogenic transcription factors.

## METHODS

### Human subjects: sample collection and group division based on BMI

This study complies with all relevant ethical guidelines approved by the Research Ethics Committee of Federal University of São Paulo (Research Ethics Committee/Federal University of São Paulo project no. 0268/2022). It also received approval through the Institutional Consent Process of the Rede D'Or São Luiz Hospitals. All participants provided written informed consent. Serum samples and vWAT (omental region from discardable amounts) were taken from male patients (*n* = 16, 30–50 years old; Table 1) undergoing elective gastric or bariatric surgery at Rede D'Or São Luiz Hospitals. Patients who were smokers; had genetic diseases; used drugs; used hormones such as insulin, glucocorticoids, or medications affecting WAT cell morphology and function; and lost weight in the last 3 months were excluded. Based on BMI, patients were grouped as follows: Group I, which includes individuals with normal weight or overweight (BMI up to 29.9 kg/m^2^): Group II, which includes individuals with class I or class II obesity (BMI between 30 and 39.9 kg/m^2^); and Group III, which includes individuals with class III obesity (BMI 40 kg/m^2^). Briefly, 5 to 15 g of WAT, in addition to plasma, was collected, packed in a sterile 50‐mL Falcon tube (Thermo Fisher Scientific Inc.) with phosphate‐buffered saline (PBS) and and antibiotics (1% penicillin/streptomycin, Gibco BRL), stored on ice, and sent to the laboratory for processing within 2 h, as described in Bellei et al. [19].

**TABLE 1:** Mean plasma biomarkers, BMI, age, and adipocyte volume among study participants.

	Group I,	Group II,	Group III,
	normal/overweight	obesity class I‐II	obesity class III
Fasting glucose, mg/dL	96.3 ±2.4	99.5 ± 15	99 ± 3.1
Triacylglycerols, mg/dL	138.2 ± 9.6	166.9 ± 27	292.5 ± 46*^#^
NEFA, μM/L	209 ± 51	230 ± 60	474 ± 120*
Total cholesterol, mg/dL	189.7 ± 7.5	216.9 ± 43	184.5.9 ± 26
HDL cholesterol, mg/dL	64.8 ± 3.8	54.4 ± 8.2	57.8 ± 9.2
Plasma endotoxin, EU/mL	2 ± 0.5	2.5 ± 0.4	5.7 ± 0.9*^#^
Circulating TNF‐α, pg/mL	0.035 ± 0.003	0.042 ± 0.003	0.037 ± 0.001
Circulating MCP‐1, pg/mL	29.2 ± 3.13	40.5 ± 13*	49.2 ± 8.2*
Circulating leptin, ng/mL	5.2 ± 0.9	12.6 ± 2.5*	17.1 ± 2.2*
Age, y	41.3 ± 4.3	48.2 ± 8.4	34.8 ± 5.3
BMI, kg/m^2^	26.9 ± 0.5	34.8 ± 5.3*	44.3 ± 6.1*^#^
Adipocyte volume, pL	320.7 ± 107	823.7 ± 140*	757.2 ± 107*

*Note*: Mean ± SEM, *p < 0.05 vs. Group I; ^#^ *p* < 0.05 vs. Group II (one‐way ANOVA and Tukey posttest).

Abbreviations: EU, endotoxin unit; HDL, high‐density lipoprotein; MCP‐1, monocyte chemoattractant protein‐1; NEFA, nonesterified fatty acids.

### Serum dosages

The blood was centrifuged at 3000*g* for 20 min at 4°C and the serum was stored at −80°C. Concentrations of total cholesterol and fractions, free fatty acids (nonesterified fatty acids), triacylglycerols, and fasting glucose were measured in serum using commercial kits (Labtest Diagnóstica; Wako Chemicals; and Quantikine M, R&D Systems, Inc.). Circulating lipopolysaccharide (LPS) was determined using a chromogenic kit for circulating endotoxins (Pierce, A39552S, Thermo Fisher Scientific). The concentrations of CCL2, tumor necrosis factor‐α (TNF‐α) and leptin (LEP) in the culture supernatant were determined using specific enzyme‐linked immunosorbent assay (ELISA) kits (Quantikine M).

### Adipose tissue processing for cell isolation

After dissection and fragmentation of vWAT, the samples were divided into two parts: 1) for analysis in the whole vWAT (stored in liquid nitrogen for later processing) and 2) placed in digestion buffer (DMEM/HEPES 20mM/bovine serum albumin [BSA] 4%, collagenase II [MilliporeSigma] 4%; 1.0 mg/mL, pH 7.40) so that the cells could be isolated by digesting the tissue with collagenase [20]. Incubation was performed for 10 to 15 min at 37°C in a water bath with orbital shaking (130 rpm). Samples were then transferred to 50‐mL Falcon tubes and the volume completed to 25 mL of EHB buffer (25mM Earle's/HEPES salts, 1% BSA, 1mM sodium pyruvate, without glucose; pH 7.45, 37°C). The filtrate was centrifuged (400*g*, 1 min) and divided into the following two fractions: 1) the upper supernatant containing isolated adipocytes (used for morphometric analysis and in another group project) and 2) the stromal cell fraction containing ASCs subjected to centrifugation (1500*g* for 5 min). The pellet was washed with EHB buffer and centrifuged twice more. It was then resuspended in culture medium (DMEM Han's F‐12, supplemented with 10% fetal bovine serum [FBS] and 1% penicillin‐streptomycin), seeded into T‐25 culture flasks (Corning Inc.) with 10 mL of medium, and maintained in a 5% CO_2_ incubator at 37°C. The medium was replaced every 2 days until the cultures reached 70% to 80% confluence. Afterward, the medium was removed, and cells were washed with PBS. The final step for ASC isolation was selecting the adherent stromal cell fraction population, considered passage “0” (P0). The cells were trypsinized, resuspended in the same incubation medium, redistributed into more culture flasks for cell expansion (the medium was renewed every 2 days), and maintained until they again reached 70% to 80% confluence (P1).

After confirming ASC isolation through immunophenotyping (with >95% purity for CD73 + CD90 + CD31 − CD45 − cells, as previously characterized [21]), the cells were either subjected to total RNA extraction for reverse transcriptase‐polymerase chain reaction (RT‐PCR) analysis or plated for in vitro treatment and proliferation studies.

### Morphometric analysis of adipocytes

For morphometric analysis, aliquots of the cell suspension were evaluated under an optical microscope with a graduated eyepiece to measure the average cell diameter (resulting from the measurement of 100 cells). Based on the average cell diameter and assuming that the isolated adipocyte is spherical, the cellular volumes were calculated as previously described [22].

### ASC proliferation potential

Proliferation was estimated by “growth curve analysis” of ASCs, using the protocol described by Capes‐Davis et al. [23]. ASCs at P1 were cultured in 100‐mm culture dishes (Corning) and maintained in DMEM/Ham's F‐12 (1:1) supplemented with 10% FBS and 1% penicillin‐streptomycin in a 5% CO_2_ incubator at 37°C. When they reached ~80% confluence, cells were trypsinized and reseeded into 12‐well plates (Corning) in equal cell numbers (5 × 10^3^ cells/well), based on the cell concentration estimated in a Neubauer chamber. The cells were collected daily for nine consecutive days.

To isolate ASCs, 500 μL of PBS was used for washing, followed by 200 μL of trypsin for 3 min, neutralized with 10% culture medium. The cells were counted in a Neubauer chamber using 0.4% trypan blue to distinguish viable cells, with nonviable cells having a blue‐stained nucleus. The logarithm of the cell count for each day of ASC cultivation was calculated and plotted on a linear graph, from which the growth curve for each culture (patient) was derived. This curve identified the lag, logarithm, plateau (stationary), and death phases, reflecting cell dynamics and representing, respectively, the adaptation, exponential growth (in which proliferation exceeds cell death), and stabilization phases (when proliferation equals death due to space limitations—contact inhibition) [23].

Additionally, the culture medium from the 9 days of cell growth was collected for quantification of the cytokines CCL2 and TNF‐α with their expression calculated per 5 × 10^3^ cells.

### RNA extraction and real‐time PCR

Total RNA was extracted with TRIzol Reagent (Invitrogen), according to the instructions by the provider. The quantity and quality of the RNA produced were verified by spectrophotometry (260 nm) in a NanoDrop (Thermo Fisher Scientific). Complementary DNA (cDNA) was synthesized from the treated RNA. Gene expression was evaluated by real‐time PCR and the analysis of the results obtained was carried out using the 2^(−∆∆Ct) method [24]. Data are expressed as the ratio between the expression of the target gene and housekeeping gene (glyceraldehyde‐3‐phosphate dehydrogenase [*GAPDH*]). Primers used the following: CREB–binding lysine acetyltransferase (*CREBBP*; also known as *CBP*; 5′‐3′sense: GAAACCAACAAACCATCCTGG; 5′‐3′antisense: CATTGGATTATTTCCCAGGG); EP300 lysine acetyltransferase (*EP300*; 5′‐3′sense: TGCAGGCATGGTTCCAGTTT; 5′3′antisense: AGGTAGAGGGCCATTAGA AGTCA); enhancer of zeste 2 polycomb repressive complex 2 subunit (*EZH2*; 5′‐3′sense: GCTGGAATCAAAGGA TACAGACA; 5′‐3′antisense: GACACCGAGAATTTGCTTCAG); lysine demethylase 6A (*KDM6A*; 5′‐3′sense: GAGGGAAGCTCTCATTGCTG; 5′‐3′antisense: AGATGAGGCGGATGGTAATG); lysine demethylase 6B (*KDM6B*; 5′‐3′sense: CTCAACTTGGGCCTCTTCTC; 5′‐3′antisense: GCCTGTCAGATCCCAGTTCT); and *GAPDH* (5′‐3′sense: GTCTCCTCTGACTTCAACAGCG; 5′‐3′antisense: ACCACCCTGTTGCTGTAGCCAA).

### Statistical analysis

Pearson correlation coefficient (*r*) was used to assess correlations. Data were analyzed using one‐way ANOVA, followed by Tukey posttest for comparisons between groups or *t* test (where appropriate). Results are expressed as mean (SEM). Differences were considered significant for *p* values < 0.05. Statistical analysis was performed using GraphPad Prism software version 9.1.2.

## RESULTS

### Patients and obesity plasma markers

The average plasma values for the respective groups are described in Table 1. As expected, patients with obesity (Groups II and III) showed significant elevations in plasma levels of triglycerides, nonesterified fatty acids, and inflammatory markers such as CCL2/MCP‐1, TNF‐α, plasma endotoxin (LPS), and LEP, along with a very significant increase in adipocyte size. The vWAT from these patients was collected and processed as described in *Methods*, and mature adipocytes were isolated and measured morphometrically to assess their size.

### Correlation among BMI, the size of adipocytes extracted from vWAT, and plasma biochemical parameters

**FIGURE 1:**
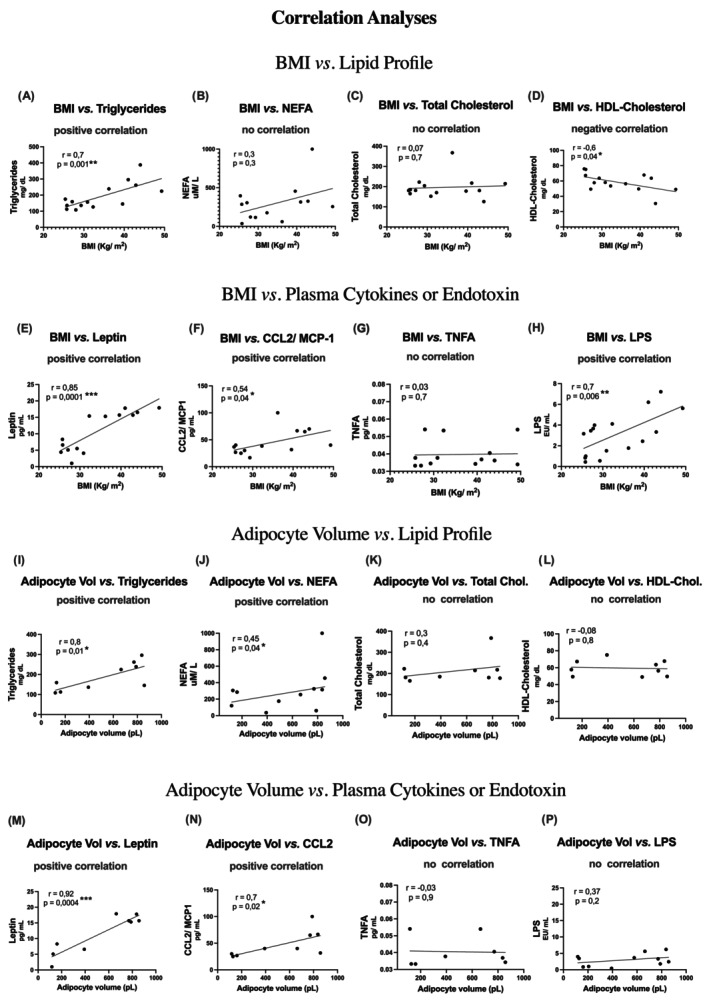
Correlation analysis between BMI or the size of adipocytes (extracted from vWAT) with plasma biochemical parameters. (A) BMI vs. plasma triglycerides; (B) BMI vs. plasma NEFA; (C) BMI vs. plasma total cholesterol; (D) BMI vs. plasma HDL cholesterol; (E) BMI vs. plasma leptin; (F) BMI vs. plasma CCL2/MCP‐1; (G) BMI vs. plasma TNF‐α; (H) BMI vs. plasma LPS; (I) adipocyte volume vs. plasma triglycerides; (J) adipocyte volume vs. plasma NEFA; (K) adipocyte volume vs. plasma total cholesterol; (L) adipocyte volume vs. plasma HDL cholesterol; (M) adipocyte volume vs. plasma leptin; (N) adipocyte volume vs. plasma CCL2/MCP‐1; (O) adipocyte volume vs. plasma TNF‐α; and (P) adipocyte volume vs. plasma LPS. Pearson correlation coefficient (*r*) and significance (*p*) values are indicated above each plot. *p < 0.05, **p < 0.001, and ***p < 0.0001. CCL2, C‐C motif chemokine 2; HDL, high‐density lipoprotein; NEFA, nonesterified fatty acids; vWAT, visceral white adipose tissue.

Correlating the average plasma values with the patients' BMI or the size of the adipocytes in WAT, we found some interesting correlations and confirmed in our studies those already described in the literature (Figure 1). We highlight positive correlations between BMI and plasma levels of triacylglycerols, LEP, CCL2, and LPS (Figure 1A,E,F,H). We also found strong positive correlations between visceral adipocyte volume and triacylglycerols, nonesterified fatty acids, LEP, and CCL2 (Figure 1I,J,M,N). On the other hand, we observed a negative correlation between BMI and HDL cholesterol (Figure 1D).

### Proliferation potential and inflammatory secretion profile of vWAT ASCs from patients with different BMI

**FIGURE 2:**
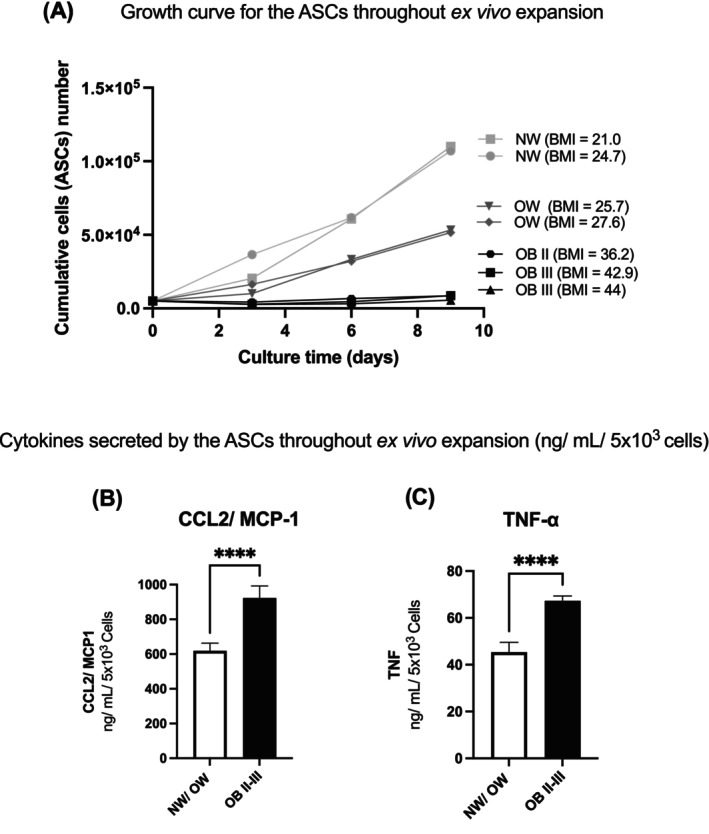
Proliferation potential and inflammatory secretion profile of ASCs extracted from vWAT of patients with different BMI values. ASCs were plated in a 12‐well plate after the first passage of in vitro culture, at a concentration of 5 × 10^3^ cells/well. Cells were collected daily for nine consecutive days and counted in a Neubauer chamber using 0.4% trypan blue dye to identify viable cells. All medium was collected to quantify cytokines secreted over 9 days, normalized by the number of cells present at the end in nanograms/milliliters/5 × 10^3^ cells, quantified by specific ELISA kits. (A) Growth curve of ASCs extracted from vWAT of patients with different BMI. (B) CCL2/MCP‐1 and (C) TNF‐α secreted in the medium. Experiments were performed in triplicate. ASC samples were grouped by patients' BMI into two groups: NW/OW and OB II‐III. Mean (SEM); ****p < 0.00001. ASC, adipose‐derived stem cell; CCL2, C‐C motif chemokine 2; NW, normal weight; OW, overweight; OB II, obesity class II; OB III, obesity class III; vWAT, visceral white adipose tissue.

ASCs from WAT of patients with different BMI values were assessed for their cell proliferation potential. To this end, we performed a growth curve analysis by culturing ASCs after the first passage (P1) of in vitro culture over nine consecutive days. We found that ASCs from patients with higher BMI exhibit a lower rate of cell expansion, as assessed at different time points (days 3, 6, and 9; Figure 2A). The results show less growth in individuals with higher BMI until day 3, followed by an exponential growth phase between days 3 and 9, reaching a growth plateau on day 9. Thus, patients in the normal weight/overweight group exhibited a significantly higher proliferation potential, reaching ~6.2 times the growth observed in the group with obesity.

The entire culture medium, in which the cells proliferated, was collected for the quantification of the cytokines CCL2 and TNF‐α secreted over a 9‐day period. Cytokine expression was normalized by the number of cells present at the end of the culture and calculated to be expressed per 5 × 10^3^ cells. The experiments were conducted in triplicate using vWAT samples from patients grouped by BMI into two categories: normal weight/overweight and obesity class II‐III. When comparing the average cytokine levels among the groups, we found that the obesity class II‐III group showed a significantly higher expression of CCL2/MCP‐1 (Figure 2B) and TNF‐α (Figure 2C), despite having a considerably lower cell count compared to the normal weight/overweight group.

### Expression of genes encoding histone modifiers H3K27 and inflammatory cytokines in human vWAT and isolated ASCs

**FIGURE 3:**
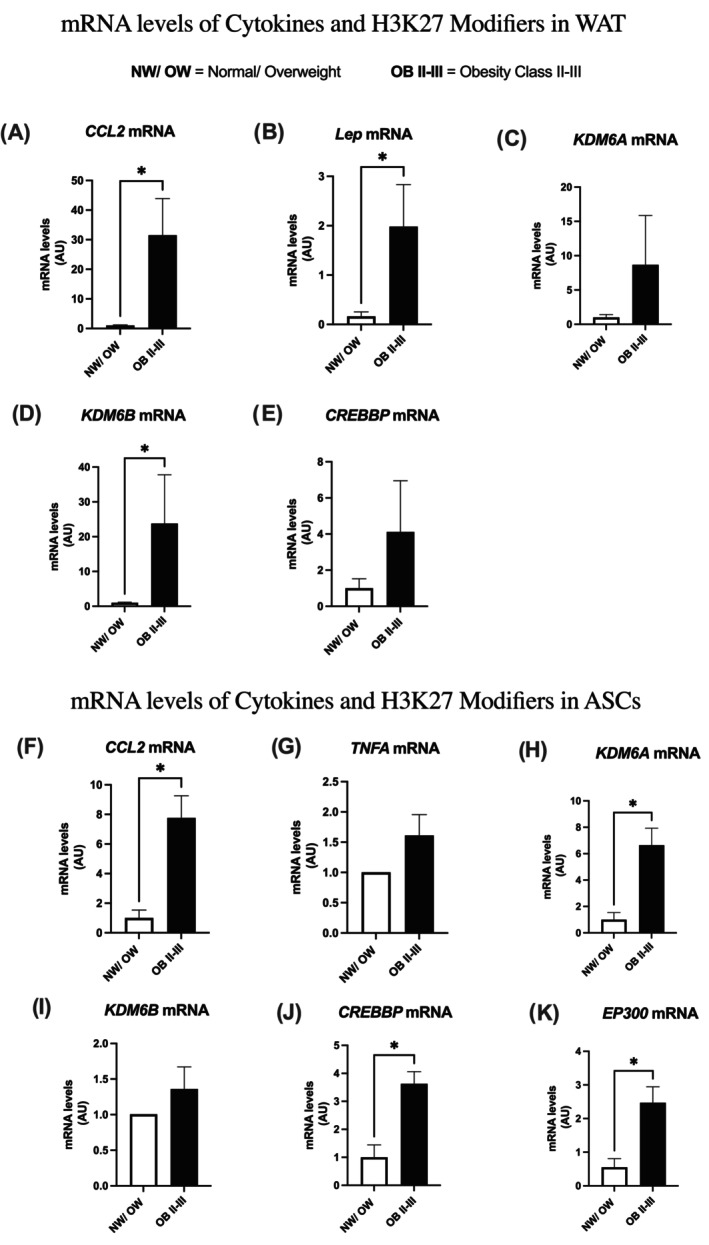
Expression of genes encoding inflammatory cytokines and histone modifiers (histone 3 lysine 27 [H3K27]) in human vWAT and ASCs. (A) *CCL2/MCP‐1* in vWAT, (B) *LEP* in vWAT, (C) KDM6A in vWAT, (D) *KDM6B* in vWAT, (E) *CREBBP* in vWAT, (F) *CCL2/ MCP‐1* in ASCs, (G) *TNF*A in ASCs, (H) *KDM6A* in ASCs, (I) *KDM6B* in ASCs, (J) *CREBBP* in ASCs, and (K) *EP300* in ASCs. Total RNA was extracted and subjected to RT‐PCR. Target gene expression was normalized by constitutive *GAPDH*. Mean (SEM); *p < 0.05. (4–6 patients per group, performed in triplicate). ASC, adipose‐derived stem cell; CCL2, C‐C motif chemokine 2; CREBBP, CREB–binding lysine acetyltransferase; EP300, EP300 lysine acetyltransferase; H3K27, histone 3 lysine 27; LEP, leptin; KDM6A, lysine‐specific demethylase 6A; KDM6B, lysine‐specific demethylase 6B; NEFA, nonesterified fatty acids; vWAT, visceral white adipose tissue.

The vWAT samples, along with isolated ASCs (after the establishment of P1 in primary culture), were also processed for extraction of total RNA and analysis by real‐time RT‐PCR for the transcripts of the main H3K27 modifiers (EZH2, KDM6A, KDM6B, EP300, and CREBP/CBP). We also assessed the expression of the proinflammatory cytokines *CCL2/MCP‐1* and *TNF*A mRNA in these samples. The results, showing the total content of these transcripts in WAT (whole tissue) or ASCs from different patients grouped by BMI, are presented in Figure 3.

We observed an increase in the expression of genes encoding CCL2, LEP, and the demethylase KDM6B (Figure 3A,B,D) in vWAT of patients with class II to III obesity, compared to the normal weight/overweight group. In the vWAT progenitor cells (ASCs), there was a substantial increase in CCL2 (Figure 3F) and an increase in the expression of *KDM6A* (Figure 3H), as well as the acetylases CREBBP and EP300 (Figure 3J,K) in the patients with obesity. On the other hand, no statistically significant difference was observed for the other transcripts evaluated.

### Correlation between BMI or H3K27 acetylase transcripts (EP300 and CREBBP) and key adipogenesis regulators expressed in vWAT and ASCs

The potential of ASCs to differentiate into adipocytes was also evaluated by assessing the expression of transcripts of key regulators related to early adipogenesis in both vWAT and ASCs. Based on the obtained values, correlation analyses were performed.

**FIGURE 4:**
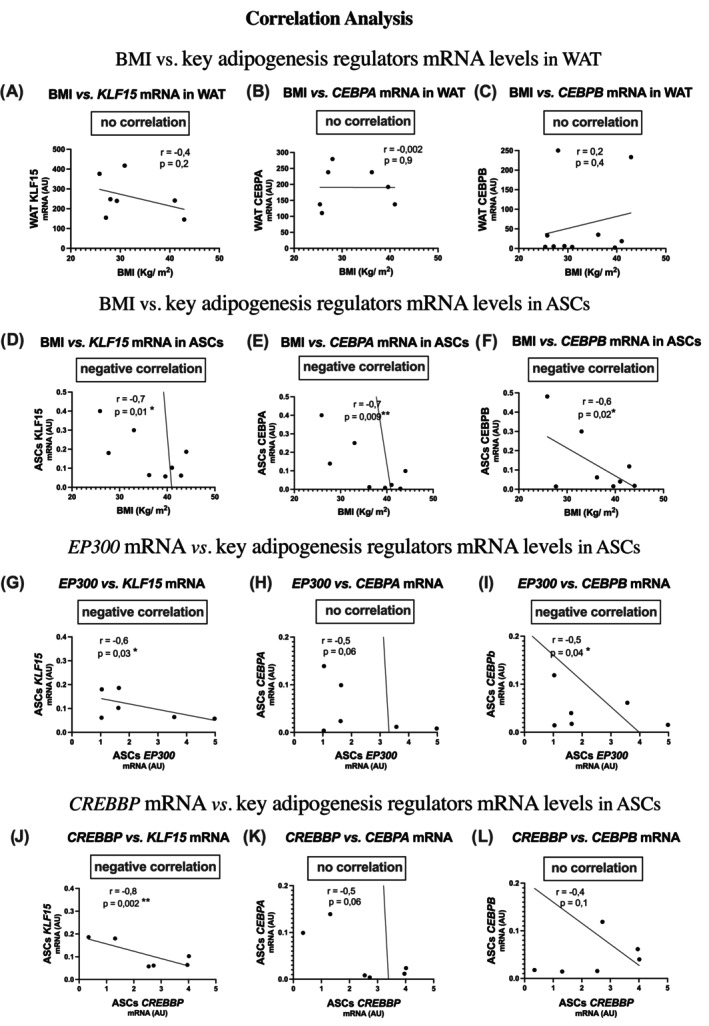
Correlation analysis between BMI or H3K27 acetylase transcripts (EP300 or CREBBP) and transcripts of key regulators related to early adipogenesis, expressed in vWAT and (ASCs. (A) BMI vs. *KLF15* in vWAT, (B) BMI vs. *CEBPA* in vWAT, (C) BMI vs. *CEBPB* in vWAT, (D) BMI vs. *KLF15* in ASCs, (E) BMI vs. *CEBPA* in ASCs, (F) BMI vs. *CEBPB* in ASCs, (G) *EP300* vs. *KLF15*, (H) *EP300* vs. *CEBPA*, (I) *EP300* vs. *CEBPB*, (J) *CREBBP* vs. *KLF15*, (K) *CREBBP* vs. *CEBPA*, and (L) *CREBBP* vs. *CEBPB*. Pearson correlation coefficient (*r*) and significance (*p*) values are indicated above each plot. *p < 0.05, **p < 0.001. ASC, adipose‐derived stem cell; CEBPA, CCAAT/enhancer‐binding protein‐α; CEBPB, CCAAT/enhancer‐binding protein‐β; CREBBP, CREB–binding lysine acetyltransferase; EP300, EP300 lysine acetyltransferase; H3K27, histone 3 lysine 27; KLF15, Krueppel‐like factor 15; vWAT, visceral white adipose tissue.

No correlation was found between BMI and key regulators of adipogenesis in vWAT (Figure 4A–C). However, significant negative correlations were observed between BMI and Krueppel‐like factor 15 *(KLF15)*, CCAAT/enhancer‐binding protein‐α *(CEBPA)*, and CCAAT/enhancer‐binding protein‐β *(CEBPB)* mRNA levels in ASCs (Figure 4D,E), suggesting impaired adipogenesis with higher BMI.

We also observed several negative correlations between H3K27 acetylase transcripts and mRNA levels of key adipogenesis regulators. Specifically, *EP300* was negatively correlated with *KFL15* and *CEBPB* mRNA (Figure 4G,I), whereas no correlation was found between *EP300* and *CEBPA* mRNA levels (Figure 4H). Regarding *CREBBP*, a negative correlation was observed with *KFL15* (Figure 4J), whereas no differences were found in relation to *CEBPA* and *CEBPB* mRNA levels (Figure 4K,L).

### Correlation between plasma leptin and CCL2 and in vitro effects of leptin on ASCs

**FIGURE 5:**
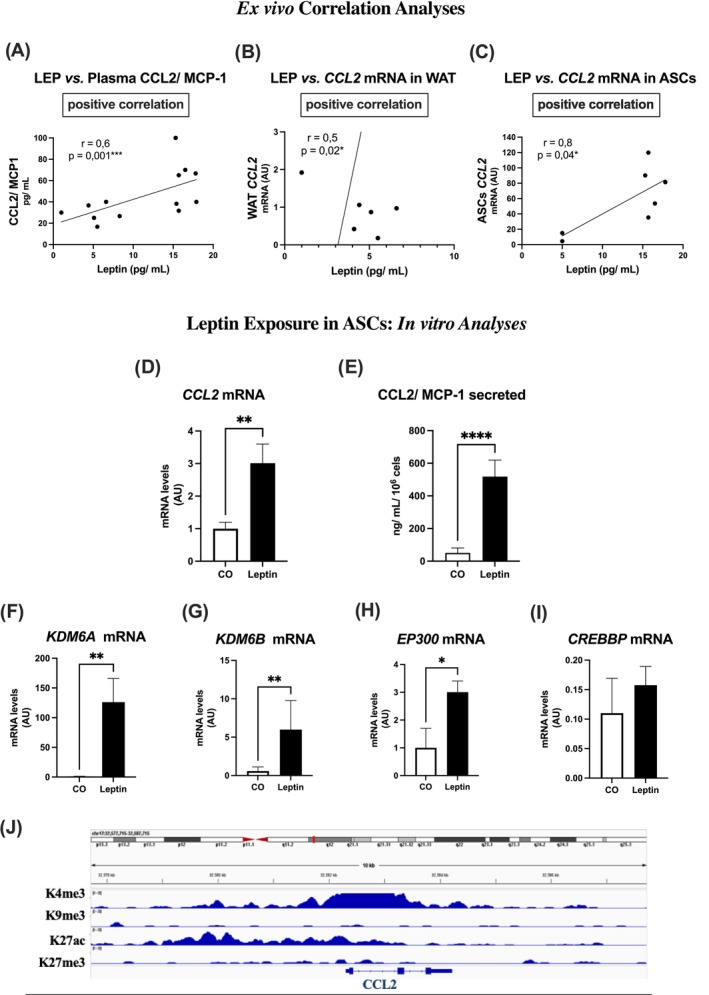
Correlations between plasma leptin and (A) plasma CCL2/MCP‐1, (B) *CCL2/MCP‐1* transcript in vWAT, and (C) *CCL2/MCP‐1* transcript in ASCs. Pearson correlation coefficient (*r*) and significance (*p*) values are indicated above each plot. Leptin effects on ASCs in vitro, including (D) *CCL2* transcript expression and (E) CCL2 protein. Transcripts encoding histone modifiers: (F) KDM6A, (G) KDM6B, (H) EP300, and (I) CREBBP. Human visceral ASCs were stimulated with leptin (100 ng/mL) for 24 h. Secreted protein quantification was performed by ELISA and mRNA transcript analysis by RT‐PCR, normalized to the constitutive gene (*GAPDH*). Values are expressed as mean (SEM); *n* = 4–6. *p < 0.05, **p < 0.001, ***p < 0.0001, ****p < 0.00001. (J) Analysis of *CCL2* gene locus enrichment for H3K4me3, H3K9me3, H3K27ac, and H3K27me3 histone marks in human ASCs isolated from abdominal subcutaneous white adipose tissue of pear‐shaped premenopausal women showing abundance of transcriptional activation marks (H3K4me3 and H3K27me3; NCBI GEO dataset GSE224770 and [25]). ASC, adipose‐derived stem cell; CCL2, C‐C motif chemokine 2; CREBBP, CREB–binding lysine acetyltransferase; EP300, EP300 lysine acetyltransferase; H3K27, histone 3 lysine 27; H3K27ac, acetylated H3K27; H3K27me3, trimethylation of histone H3 at lysine 27; H3K4me3, trimethylation of lysine 4 on the histone H3 protein; H3K9me3, epigenetic modification to the DNA packaging protein histone H3; KDM6A, lysine‐specific demethylase 6A; KDM6B, lysine‐specific demethylase 6B; vWAT, visceral white adipose tissue. [Color figure can be viewed at wileyonlinelibrary.com]

We observed a highly significant correlation between plasma leptin levels and the expression of CCL2/MCP‐1, both in circulation (Figure 5A) and in vWAT and ASCs (mRNA transcripts; Figure 5B,C).

To further explore these ex vivo findings and better understand the role of leptin in the expression of this important chemokine by adipose progenitor cells, ASCs isolated from normal weight patients were directly exposed to leptin (in vitro) for 24 hours. We then evaluated CCL2/MCP‐1 secretion and gene expression. The response was a substantial increase in cytokine secretion by ASCs (Figure 5D,E), accompanied by an increase in the expression of transcripts encoding the demethylases KDM6A and KDM6B, as well as the acetylase EP300 (Figure 5G–I). This, in turn, could increase the content of acetylated H3K27 (H3K27ac) in the nucleus of these cells. Using the Integrative Genomics Viewer software (version 2.16.0, https://igv.org), we identified an enrichment of H3K27ac at the promoter region of CCL2/MCP‐1 in human ASCs (Figure 5J), which further supports our findings.

## DISCUSSION

The main purpose of this study was to investigate how obesity‐related plasma markers correlate with epigenetic and inflammatory changes in adipose progenitor cells, the ASCs. Consistent with the literature, we observed significant hypertrophy in adipocytes from vWAT samples with obesity (Groups II and III) and an increase in LPS and inflammatory cytokines, including leptin and CCL2/MCP‐1. These cytokines contribute to the chronic low‐grade inflammation that is a hallmark of obesity and play a key role in the development of comorbidities such as insulin resistance, hypertension, hepatic steatosis, dyslipidemia, and type 2 diabetes [26, 27]. Moreover, the recruitment of ASCs into adipogenesis is essential for healthy WAT expansion, and herein, we also demonstrated that these cells were directly impacted by increases in BMI.

To better understand the impact of obesity on ASCs, we first assessed their proliferation potential and cytokine secretion profile, besides adipogenesis potential. Our results showed that ASCs from patients with higher BMI exhibited a lower proliferation rate. However, these cells secreted significantly higher levels of CCL2/MCP‐1 and TNF‐α. This suggests that, although ASCs in individuals with obesity proliferate at a slower rate, they have an enhanced inflammatory profile. These findings corroborate other studies, which have shown that ASCs with obesity demonstrate a lower proliferation rate [28] and an enhanced proinflammatory phenotype [29]. Additionally, a recent review by Przywara et al. [30] summarized that ASCs in obesity are defective in various functionalities and properties, including proliferation, anti‐inflammatory properties, cell aging, angiogenesis, and exosome size and number [17].

Our results also provide novel insights into the impact of obesity on adipogenesis, particularly regarding the expression of key adipogenic regulators and the role of histone modifications in ASCs. The lack of correlation between BMI and key regulators of adipogenesis in vWAT suggests that the impairment of adipogenesis in WAT might occur through mechanisms beyond the expression of these regulators, potentially involving other cellular or microenvironmental factors.

There were significant negative correlations between BMI and the mRNA levels of *KLF15*, *CEBPA*, and *CEBPB* in ASCs, which indicates that higher BMI is associated with impaired early adipogenesis. This finding aligns with previous studies indicating that obesity can hinder ASC differentiation, disrupting adipocyte formation [17]. KLF15, CEBPA, and CEBPB are crucial for the early stages of adipocyte differentiation, and their downregulation in ASCs may reflect a shift away from adipogenic potential as obesity progresses. In agreement, studies using animal models have shown that the expression of adipogenesis‐related genes in ASCs from mice with obesity is significantly reduced, as reviewed by Yang et al. [31].

Furthermore, our results suggest that histone acetylation plays a role in modulating adipogenesis, as indicated by negative correlations between H3K27 acetylase transcripts (*EP300* and *CREBBP*) and key adipogenic regulators. Specifically, the negative correlations between *EP300* and *KLF15*, as well as between *EP300* and *CEBPB*, suggest that acetylation by *EP300* may inhibit the expression of these factors. Similarly, the negative correlation between *CREBBP* and *KLF15* supports the idea that histone acetyltransferases, such as *EP300* and *CREBBP*, are involved in the epigenetic regulation of adipogenesis. These findings are consistent with studies showing that histone acetylation can influence gene expression during adipocyte differentiation [32]. The absence of correlations between *EP300* and *CEBPA* mRNA levels, and between *CREBBP* and *CEBPA/CEBPB*, suggests that histone acetylation may specifically affect certain transcription factors in adipogenesis, rather than globally influencing all adipogenic markers.

The literature highlights that obesity‐related complications are closely linked to the expansion of WAT, characterized by hypertrophic adipocytes and the infiltration of inflammatory cells, particularly M1 macrophages, which increase CCL2 expression, promoting inflammation and insulin resistance, a precursor to type 2 diabetes [27, 33, 34]. Although healthy WAT expansion involves adipose precursor cell recruitment, metabolically unhealthy obesity, marked by limited sWAT storage, results in fat accumulation in ectopic tissues and vWAT depots [35]. Our findings show that obesity enhances CCL2 expression in ASCs, alongside increased expression of H3K27 modifiers, key epigenetic regulators linked to gene activation in WAT stromal cells. Specifically, we observed elevated levels of *KDM6A*, *CREBBP*, and *EP300* histone modifiers in ASCs from patients with obesity class II‐III.

We also found significant correlations among leptin levels, BMI, and adipocyte size. Specifically, leptin levels correlated positively with both CCL2/MCP‐1 levels in plasma and vWAT, as well as CCL2 expression in ASCs. This suggests that leptin contributes to the inflammatory milieu in obesity by upregulating CCL2, which in turn facilitates macrophage infiltration into WAT. We then hypothesized that leptin, highly elevated in patients with obesity and, in our studies, markedly correlated with higher CCL2/MCP‐1 expression in both plasma and vWAT, as well as in ASCs, increases the expression of this chemokine in ASCs by regulating H3K27 acetylation.

To investigate, we assessed the direct effect of leptin on H3K27 modifiers and CCL2 secretion by ASCs, simulating the leptin‐rich environment of obesity in vitro. Leptin treatment led to a significant increase in both CCL2 gene expression and protein secretion in ASCs isolated from patients, suggesting a direct action of leptin on these cells. This effect was further associated with increased levels of *KDM6A* and *KDM6B*, along with elevated *EP300* expression, highlighting the epigenetic changes induced by leptin. Using Integrative Genomics Viewer, we detected H3K27ac enrichment at the CCL2/MCP‐1 promoter region in human ASCs, further supporting the idea that H3K27 acetylation mediates the effect of leptin on MCP‐1 expression. Additionally, the involvement of H3K27 acetylation in the regulation of CCL2 has been further validated in recent studies. Akhter et al. [14] demonstrated the critical role of H3K27ac in the positive regulation of the CCL2 promoter in LPS‐stimulated mononuclear cells. Although we did not investigate the effect of LPS on ASCs in our study, we observed similar trends in response to LPS stimulation in our recent work [21, 36] because we found increased CCL2 expression and upregulation of *KDM6B* and *EP300* in ASCs exposed to LPS. These results, in conjunction with our findings on leptin, suggest that histone modifications, particularly H3K27 acetylation, are key regulators of MCP‐1 expression in ASCs during both obesity and inflammatory conditions. Further studies, particularly chromatin immunoprecipitation (ChIP)‐RT‐PCR, are under way to confirm these findings and to further elucidate the role of histone modifications in CCL2 regulation.

In conclusion, our findings suggest that leptin, through its effects on H3K27 epigenetic changes, plays a pivotal role in regulating CCL2/MCP‐1 expression in ASCs, revealing a novel mechanism by which leptin may contribute to WAT inflammation and the pathophysiology of obesity. The interplay among histone acetylation, demethylation, and gene regulation provides valuable insights into the molecular pathways underlying obesity‐related inflammation, highlighting potential therapeutic targets for alleviating inflammatory responses. Additionally, altered epigenetic regulation and impaired proliferation in ASCs appear to contribute to reduced adipogenesis potential in obesity, further propagating chronic inflammation in vWAT. Our study underscores the role of histone modifications and key adipogenic transcription factors in the impairment of adipogenesis in obesity, pointing to future research on the mechanisms of H3K27 modification and its impact on adipogenesis as crucial for understanding the molecular basis of obesity and its associated metabolic disorders. It is important to mention that our data were obtained from male patients, as the exclusion of female participants helped control for gender‐specific hormonal influences on adipose tissue metabolism [37]. Although this approach enhances internal validity, it may limit the generalizability of our findings. Future studies should explore these differences to better understand sex‐specific metabolic responses in obesity‐related conditions.

## AUTHOR CONTRIBUTIONS

Conceptualization, methodology, formal analysis, and investigation were done by Andressa França Sousa Bispo, Jussara de Jesus Simao, Miguel Ambrizzi Moraes, André Valente Santana, Paula Volpe, and Maria Isabel Cardoso Alonso‐Vale. Visualization, validation, methodology, and investigation were done by Victor Tadeu Gonçalves Plata and Ana Beatriz Marques Abel. The writing of the original draft was done by Andressa França Sousa Bispo and Jussara de Jesus Simao. The review and editing were done by Lucia Maria Armelin‐Correa, Monica Marques Telles, and Maria Isabel Cardoso Alonso‐Vale. Visualization was also done by Andressa França Sousa Bispo, Jussara de Jesus Simao, Miguel Ambrizzi Moraes, and Maria Isabel Cardoso Alonso‐Vale. Supervision was done by Maria Isabel Cardoso Alonso‐Vale. Project administration was done by Andressa França Sousa Bispo, Jussara de Jesus Simao, Miguel Ambrizzi Moraes, and Maria Isabel Cardoso Alonso‐Vale. Funding acquisition was done by Maria Isabel Cardoso Alonso‐Vale. All authors have read and agreed to the published version of the manuscript.

## CONFLICT OF INTEREST STATEMENT

The authors declared no conflicts of interest.

## Data Availability

The data that support the findings of this study are available from the corresponding author upon reasonable request.
